# Selection of Suitable Organic Amendments to Balance Agricultural Economic Benefits and Carbon Sequestration

**DOI:** 10.3390/plants13172428

**Published:** 2024-08-30

**Authors:** Hui Cao, Junming Liu, Shoutian Ma, Xiaolei Wu, Yuanyuan Fu, Yang Gao

**Affiliations:** 1 Institute of Farmland Irrigation, Chinese Academy of Agricultural Sciences, Xinxiang 453002, China; 17809268435@163.com (H.C.); liujunming0225@163.com (J.L.); wuxiaolei@caas.cn (X.W.); fyy2016060105@163.com (Y.F.); 2 Key Laboratory of Crop Water Use and Regulation, Ministry of Agriculture and Rural Affairs, Xinxiang 453002, China; 3 Graduate School of Chinese Academy of Agricultural Sciences, Beijing 100081, China; 4 Institute of Western Agriculture, Chinese Academy of Agricultural Sciences, Changji 831100, China

**Keywords:** organic amendment, nitrogen fertilizer, economic benefit, carbon emission, carbon stock

## Abstract

Long-term excessive use of fertilizers and intensive cultivation not only decreases soil organic carbon (SOC) and productivity, but also increases greenhouse gas emissions, which is detrimental to sustainable agricultural development. The purpose of this paper is to identify organic amendments suitable for winter wheat growth in the North China Plain by studying the effects of organic amendments on the economic benefits, carbon emissions, and carbon sequestration for winter wheat fields and to provide a theoretical basis for the wide application of organic amendments in agricultural fields. The two nitrogen rates were N0 (0 kg ha^−1^) and N240 (240 kg ha^−1^), and the four organic amendments were straw, manure, mushroom residue (M R), and biochar. The results showed that, compared to N0, N240 significantly increased the yield by 244.1–318.4% and the organic carbon storage by 16.7–30.5%, respectively, but increased the carbon emissions by 29.3–45.5%. In addition, soil carbon stocks increased with all three types of organic amendments compared to the straw amendment, with the biochar treatment being the largest, increasing carbon storage by 13.3–33.6%. In terms of yield and economic benefits, compared to the straw amendment, the manure and biochar amendments increased winter wheat yields by 0.0–1.5% and 4.0–13.3%, respectively, and M R slightly decreased wheat yield; only the economic benefit of the M R amendment was greater than that of the straw amendment, with an increase in economic benefit of 1.3% and 8.2% in the 2021–2022 and 2022–2023 seasons, respectively. Furthermore, according to the net ecosystem productivity (NEP), N0 was the source of CO_2_, while N240 was a sink of CO_2_. The TOPSIS results showed that N240 with a mushroom residue amendment could be recommended for increasing soil carbon stocks and economic benefits for winter wheat in the NCP and similar regions. Low-cost M R can increase farmer motivation and improve soil organic carbon, making a big step forward in the spread of organic materials on farmland.

## 1. Introduction

Food security is seriously threatened by the growing global population [1]. It is common practice to use chemical fertilizers to increase crop yields, but excessive application of nitrogen fertilizers leads to soil acidification [2], nitrogen loss [3], and reduces soil organic carbon sequestration [4], which in turn causes huge economic losses [5] and leads to the deterioration of agro-ecosystem health [6]. Therefore, how to effectively reduce economic losses and protect the health of agro-ecosystems is particularly important. As soil organic carbon has multiple functions in regulating the complex network of interactions in the soil, enhancing soil organic carbon is a key strategy for improving soil fertility [7]. Increasing soil organic carbon can improve crop yield by 23%, reduce fertilizer input by 5–7% [8], and reduce atmospheric CO_2_, and when soil organic carbon reaches the optimal level (12.7–13.4 g kg^−1^), the yield tends to remain stable [9]. Therefore, it is feasible to increase crop yield, reduce greenhouse effects, and increase soil carbon storage.

Large amounts of crop residue, industrial waste (mushroom residue) and livestock manure are generated globally every year [10,11,12], leading to enormous pressure on the environment. To improve the quality of agricultural soils and the utilization of organic materials and reduce the pressure of organic materials on the environment, the use of organic amendments is strongly recommended by researchers from different countries [13,14,15]. The application of organic amendments (OAs) is important for carbon sequestration and mitigation, but improper application of organic amendments (type and amount) can lead to different responses in the soil [16]. Compared to chemical fertilizers alone, chemical fertilizers with organic amendments increase organic carbon content by 7.6–17.3%, and chemical fertilizers with straw biochar mainly increase easily oxidizable organic carbon (EOC) and particulate organic carbon (POC), whereas chemical fertilizers with straw or soybean cake mainly increase dissolved organic carbon (DOC) and microbial biomass carbon (MBC) [17]. A meta-analysis also showed that organic amendments improved soil organic matter (SOM) by 3.6 units, with compost and biochar providing the greatest improvement in SOM and biosolids contributing the least to SOM [18], and a high N content and low C: N with the continuous application of organic amendments lead to a greater carbon sequestration [19]. Compared to compost, biochar reduced N_2_O emissions by 55%, and compared to fresh manure and compost, biochar increased organic carbon storage by 63% and 33%, respectively [20]. Meanwhile, Doblas-Rodrigo et al. [21] found that for grassland, only solid slurry fraction and late fermented solid slurry fraction significantly increased organic carbon stocks, and other organic amendments were able to keep soil organic carbon stocks unchanged. And for maize fields, most of the organic amendments decreased soil organic carbon stocks. In addition, some studies have been conducted by national researchers. For example, chemical fertilizer combined with manure significantly increased carbon emissions and had lower organic carbon content, but chemical fertilizer combined with biochar had the highest organic carbon content [22]. In order to obtain a reasonable fertilization regime with organic amendments, Zhang et al. [23] showed that long-term application of straw and manure increased yields by 6.4% and 9.9%, and greenhouse gas emissions by 25.7% and 48.6%, respectively, and combined with the net ecosystem economic prognosis (NEEB), it was found that chemical fertilizers combined with cow manure were more appropriate. Yi et al. [24] concluded that chicken manure combined with biochar can not only increase soil nutrient content and improve soil porosity, but also reduce soil heavy metal concentrations; meanwhile, Xu et al. [7] showed that organic amendments increased organic carbon stocks and crop yield, but the application of excessive organic amendments decreased the efficiency of organic carbon sequestration in infertile sandy soils. All of the above studies indicate that the selection of appropriate organic amendments is critical.

In addition, price is an important limiting factor for the widespread use of organic amendments [25]. Investigations in the Central Highlands of Kenya showed that the highest benefits of animal manure were obtained in Gatanga at USD 440.74, while the highest benefits of animal manure with fertilizer were obtained in Meru South at USD 456.25 [26]. Meanwhile, studies in rural areas of southern Italy showed that economic benefits were guaranteed when the price of vermicompost was below 0.84 EUR/kg [27]. In addition, replacing 15% and 30% of N with organic fertilizers can improve economic efficiency compared to N fertilization alone, but replacing 30% of N with organic fertilizers is a better choice in terms of long-term economic efficiency and ecology [28].

The North China Plain (NCP) is a major grain production area in China, accounting for 70% of the country’s wheat production [29]. The high-intensity rotation pattern of winter wheat and summer maize ensures food security but accelerates soil degradation by relying on better management patterns (water and fertilizer management, pest and disease control, mechanical operations, etc.), resulting in annual carbon losses of up to 77 g m^−2^ a^−1^ [30,31,32]. Therefore, it is necessary to take appropriate measures to balance soil quality and farmers’ benefits. However, current researchers have mostly focused on the effects on soil quality of one or two types of organic materials combined with chemical fertilizers, especially in terms of increasing soil organic matter and suppressing greenhouse gas emissions, ignoring the price of organic materials and the burden of labor, which actually has a negative impact on the applicability of organic materials on farmland.

Therefore, the objective of this research paper is to evaluate the effects of organic amendments on winter wheat yield, economic benefits, soil carbon emissions, soil organic carbon stocks (SOCs), and net ecosystem productivity (NEP) of winter wheat in the North China Plain. Then, the organic amendment that is beneficial to the growth of winter wheat in the North China Plain is determined, providing a theoretical basis for a wide application of organic amendments in farmland.

## 2. Materials and Methods

### 2.1. Experimental Site

**Figure 1:**
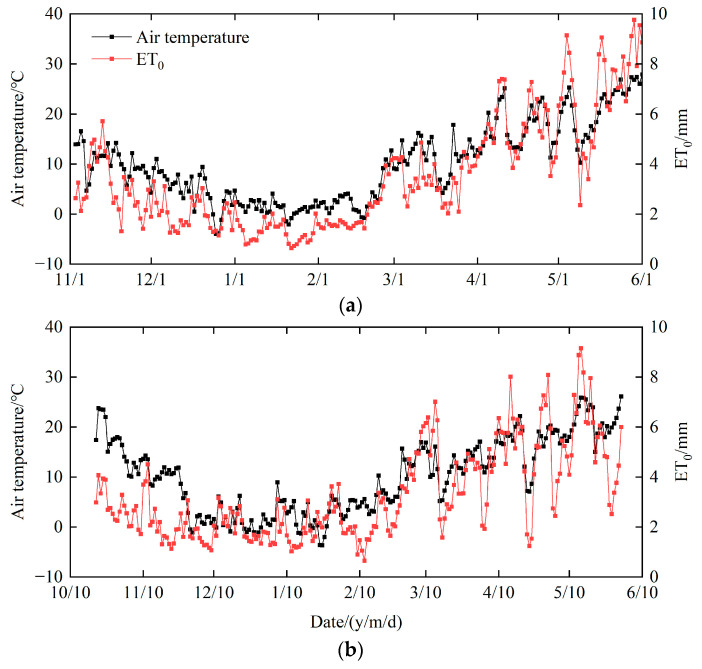
Air temperature and reference evapotranspiration (ET_0_) during the winter wheat seasons of 2021–2022 (**a**) and 2022–2023 (**b**).

A 2-year field experiment was conducted from October 2021 to June 2023 at the Xinxiang Experimental Station of the Chinese Academy of Agricultural Sciences (35.15° N, 113.80° E), located in Xinxiang Country, Henan Province, China. The area has a warm –temperate continental monsoon climate, with an average annual temperature of about 14 °C, an average rainfall of 582 mm, and a frost-free period of about 210 days. The soil type of the experimental area is loamy, and the average bulk density is 1.51 g cm^−3^. The basic chemical properties of the soil are given in Table 1. Rainfall during the two winter wheat seasons was 89 mm and 180 mm, respectively. Air temperature and reference evapotranspiration (ET_0_) are shown in Figure 1.

### 2.2. Experimental Design

**Figure 2:**
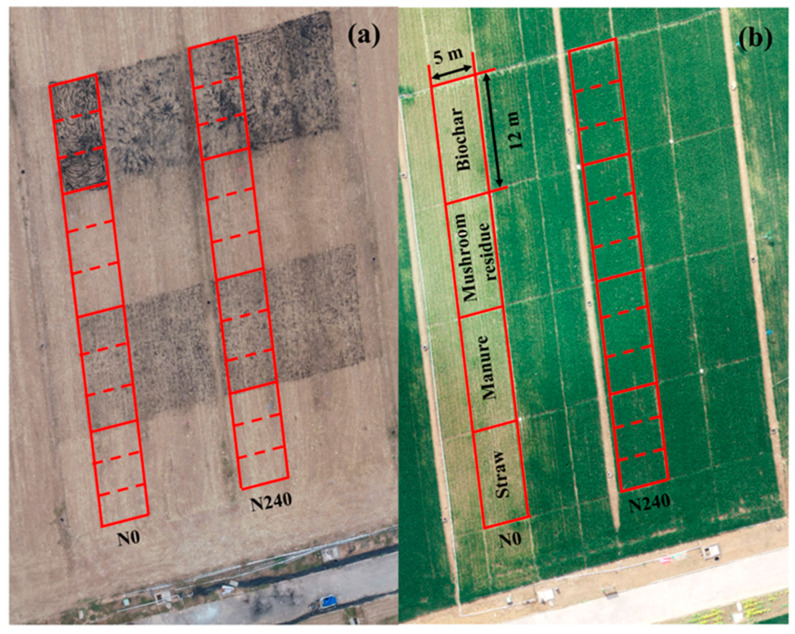
Experimental treatments and experimental plots. (Note: (**a**) represents differences among treatments before sowing; (**b**) represents differences among treatments at the jointing stage.)

The experiment consisted of four organic amendments and two nitrogen levels. The two nitrogen rates were 0 and 240 kg ha^−1^. Our previous results showed that the nitrogen rate of 240 kg ha^−1^ was an optimal rate for wheat production in the experimental region [4,33]. The four organic amendments were straw, manure, mushroom residue (M R), and biochar. The two nitrogen rates and the four organic amendments interacted as 8 experimental treatments, and the area of each treatment was 60 m^2^ with three replications (Figure 2). We measured the nitrogen concentration of maize straw and organic amendments and then obtained the total nitrogen content of the maize straw based on the biomass of the maize straw. The total nitrogen content of the organic amendments applied to the farmland was the same as that of the maize straw. All organic amendments in each plot were applied to the field before wheat sowing. The application of manure, M R, and biochar is shown in Table 2.

This experiment was carried out on the plots of a long-term fertilizer experiment, which was described in detail in [4]. The winter wheat variety was Zhoumai-22, the sowing rate was 225 kg ha^−1^, and the row spacing was 20 cm. The two winter wheat seasons were from 16 October 2021 to 1 June 2022, and from 24 October 2022 to 3 June 2023. Before sowing, 105 kg ha^−1^ of K_2_O and 120 kg ha^−1^ of P_2_O_5_ were applied in each treatment; a total of 50% of urea was applied to the soil as a basal fertilizer before sowing, and 50% urea was applied to the soil before wheat flowering (N240). The N concentration of manure, mushroom residue, and biochar were 1.71%, 9.5%, and 2.8%, respectively.

To ensure the emergence of winter wheat seedlings, an irrigation amount of 18.34 mm was applied with sprinkling irrigation on 1 November 2021 and 28 October 2022. After the winter wheat reviving stage, drip irrigation was used for wheat irrigation.

Drip irrigation consisted of drip line spacing of 60 cm, emitter spacing of 30 cm, and an emitter rate of 2.2 L h^−1^. Drip irrigation was controlled by the difference between crop evapotranspiration (ETc) and rainfall (P). That is, irrigation was applied when ETc-P = 45 mm [34].

### 2.3. Measurements

#### 2.3.1. Soil Respiration Rate (R) and Carbon Emission (CE)

After winter wheat sowing, soil collars (polyvinyl chloride tubes with an inner diameter of 20 cm and a height of 10 cm) were driven into the soil to a depth of 5 cm. Soil respiration rates were measured every 3 to 4 days using the LI-8100A automated soil CO_2_ flux measurement system (LI-COR Inc, Lincoln, NE, USA). In addition, soil respiration rates were measured for three consecutive days after rainfall, irrigation, and fertilizer application.

Soil respiration rate (*R*) [35] and carbon emission (*CE*) [36] were calculated with Equations (1) and (2).

(1) $$R=\frac{10VP_{0}(1-\frac{W_{0}}{1000})\partial C^{\prime }}{RS(T_{0}+273.15)\partial t}$$

(2) $$CE=\sum \left[(R_{N+1}+R_{N}\right)\times 0.5\times (t_{N+1}-t_{N})\times 0.1584\times 24]\times 0.2727\times 10$$

where *R* is soil respiration rate, μmol (m^2^·s)^−1^; *V* is the collar volume, cm^3^; *P*_0_ is pressure, kPa; *W*_0_ is the initial water vapor molar fraction (mmol mol^−1^); *S* is the measured soil area, cm^2^; *T* is the initial air temperature, °C; and $\frac{\partial c^{\prime }}{\partial t}$ is the initial rate of change in CO_2_ molar fraction after moisture correction, μmol mol^−1^.

*CE* is soil carbon emission, kg C ha^−1^; *N* is the sampling frequency; and *t* is the sampling time.

#### 2.3.2. Wheat Biomass and Grain Yield

The 30 sampled plants were cut from the soil surface, dried uniformly at 75 °C for approximately 4 days, and weighted for calculating the aboveground biomass after the wheat was harvested [37]. At harvest, a harvested area of 1 m^2^ in each plot was sampled for grain yield measurements. The wheat grain yield was manually threshed, sun-dried, weighed, and converted to a yield with a moisture content of 13%.

#### 2.3.3. Partial Factor Productivity of N (PFPN) and Nitrogen Agronomic Efficiency (NAE)

Partial factor productivity of N (PFPN, kg kg^−1^) and nitrogen agronomic efficiency (NAE, kg kg^−1^) were calculated with Equations (3) and (4).

*PFPN* = *Y_N_*/*N* (3)

*NAE* = *Y_N_* − *Y*_0_/*N* (4)

where Y_N_ (kg ha^−1^) is the yield of nitrogen treatment, Y_0_ (kg ha^−1^) is the yield of unapplied nitrogen treatment, and N (kg ha^−1^) is the nitrogen application rate.

#### 2.3.4. Carbon Emission Efficiency (CEE) and Ecosystem Carbon Balance

Carbon emission efficiency was calculated as follows:

*CEE* = *Y*/*CE* (5)

where CEE is the grain yield per 1 kg of carbon released, kg kg^−1^; Y is the crop grain yield, kg ha^−1^; and CE is soil carbon emission, kg C ha^−1^.

Ecosystem carbon balance was assessed by net ecosystem productivity, which was calculated as follows [36]:

*NEP* = *NPP-Rm* = *Total biomass* × 0.4 − *Rs* × 0.865 (6)

where NEP is the net ecosystem productivity (kg C ha^−1^); NPP is the net primary productivity (kg C ha^−1^), which is calculated by Total biomass × 0.4 (total biomass is the sum of aboveground biomass and underground biomass; underground biomass is calculated based on 32% of grain biomass) [38]; Rm is the soil microbial heterotrophic respiration (kg C ha^−1^), which is calculated as Rs × 0.865; and Rs is the cumulative soil respiration (kg C ha^−1^).

#### 2.3.5. Soil Organic Carbon and the Storage of Organic Carbon

Soil samples were collected at a depth of 0–40 cm using a soil auger (internal diameter 5 cm) from three replicates in each treatment after the winter wheat harvest (June 2022 and 2023). Then, the soil samples were dried, sieved, and weighed. The SOC content of the soil samples was measured using the oil bath–K_2_Cr_2_O_7_ titration method [39].

The stock of soil organic carbon was calculated with Equation (7):

*SOC_S_* = *SOC* × *BD* × *D* × 0.1 (7)

where SOC_S_ is the SOC stock (mg ha^−1^), BD is the bulk density (g cm^−3^), and D is the soil thickness (cm).

### 2.4. Economic Benefits Analysis

Economic analysis was based on differences in input and output differences. Detailed agronomic management components of both the winter wheat growing season wheat production and input and output prices based on market prices are presented in Table 3. All the prices for the agronomy are shown in Liu et al. [40].

### 2.5. TOPSIS Model

TOPSIS is a comprehensive evaluation method for multi-objective decision making for a finite number of solutions, which is ranked using ‘positive ideal solutions’ and ‘negative ideal solutions’ of the decision problem [41]. The highest-ranked solution is the optimal solution. The model has the following seven steps.

Step 1: Construct the original matrix.

(8) $${X=\left[\begin{array}{ccc} x_{11} & \cdots & x_{1n}\\ \vdots & \ddots & \vdots \\ x_{m1} & \cdots & x_{mn} \end{array}\right]}_{m\times n}$$

where *x_ij_* is the *I*th evaluation indicator for the *J*th evaluation project, and *m* = 8, *n* = 7 in the experiment.

Step 2: Normalize the original matrix. We use the polar variation method for normalization, as shown in Equations (10) and (11).

(9) $$R={\left[\begin{array}{ccc} r_{11} & \cdots & r_{1n}\\ \vdots & \ddots & \vdots \\ r_{m1} & \cdots & r_{mn} \end{array}\right]}_{m\times n}$$

(10) $$r_{ij}=\frac{x_{ij}-min\left\{x_{ij}\right\}}{max\left\{x_{ij}\right\}-min\left\{x_{ij}\right\}}$$

(11) $$r_{ij}=\frac{max\left\{x_{ij}\right\}-x_{ij}}{max\left\{x_{ij}\right\}-min\left\{x_{ij}\right\}}$$

where $max\left\{x_{ij}\right\}$ and $min\left\{x_{ij}\right\}$ are the maximum and minimum values under the same indicator *i*, and *r_ij_* is the value of the *J*th evaluation project of the *I*th indicator after unification.

Step 3: Indicator entropy.

(12) $$P_{ij}=\frac{x_{ij}}{\sum_{i=1}^{m}x_{ij}}$$

(13) $$e_{j}=-\frac{1}{\ln n}(\sum_{i=1}^{m}P_{ij}\times \ln P_{ij})$$

(14) $$w_{j}=\frac{1-e_{j}}{\sum_{j=1}^{n}\left(1-e_{j}\right)}$$

where *e_j_* is the indicator entropy, and *w_j_* is the weight of individual data.

Step 4: Construct the entropy-weight original matrix.

(15) $$Z={\left[\begin{array}{ccc} z_{11} & \cdots & z_{1n}\\ \vdots & \ddots & \vdots \\ z_{m1} & \cdots & z_{mn} \end{array}\right]}_{m\times n}$$

(16) $$z_{ij}=w_{j}\times r_{ij}$$

Step 5: Determine positive and negative ideal solutions.

Positive ideal solution:

(17) $$Z^{+}=(Z_{1}^{+},Z_{2}^{+},\ldots ,Z_{n}^{+})\;=(max\left\{z_{11},z_{21},\ldots ,z_{m1}\right\},min\left\{z_{12},z_{22},\ldots ,z_{n2}\right\},\ldots ,min\left\{z_{1n},z_{2n},\ldots ,z_{mn}\right\})$$

Negative ideal solution:

(18) $$Z^{-}=(Z_{1}^{-},Z_{2}^{-},\ldots ,Z_{n}^{-})\;=(min\left\{z_{11},z_{21},\ldots ,z_{m1}\right\},max\left\{z_{12},z_{22},\ldots ,z_{n2}\right\},\ldots ,max\left\{z_{1n},z_{2n},\ldots ,z_{mn}\right\})$$

Step 6: Calculate the Euclidean distance between each scheme and the ideal solution.

(19) $$D_{i}^{+}=\sqrt{\sum_{j=1}^{n}{\left(z_{i}^{+}-z_{ij}\right)}^{2}}$$

(20) $$D_{i}^{-}=\sqrt{\sum_{j=1}^{n}{\left(z_{i}^{-}-z_{ij}\right)}^{2}}$$

Step 7: Calculate the relative closeness of each solution.

(21) $$C_{i}=\frac{D_{i}^{-}}{D_{i}^{-}+D_{i}^{+}}$$

Finally, arrange the positions based on the $C_{i}$ values and proceed to the next step based on these positions.

### 2.6. Statistical Analysis

Microsoft Office 2021 software was used for data collection and Origin 2023b for graphing. All statistical analyses were performed using SPSS software (version 26.0, IBM Corporation, Armonk, NY, USA). Data were transformed if a Gaussian distribution was not achieved. Two-factor analysis of variance was used to examine the effects of organic amendment types on winter wheat yield, economic benefits, carbon emissions, organic carbon stock, and NEP under different nitrogen fertilizers rates. The means of the variables were compared using the LSD test (*p* < 0.05). Meanwhile, the TOPSIS model was used for comprehensive evaluation.

## 3. Results

### 3.1. Soil Respiration Rate, Soil Carbon Emission (CE) under Different Treatments

**Figure 3:**
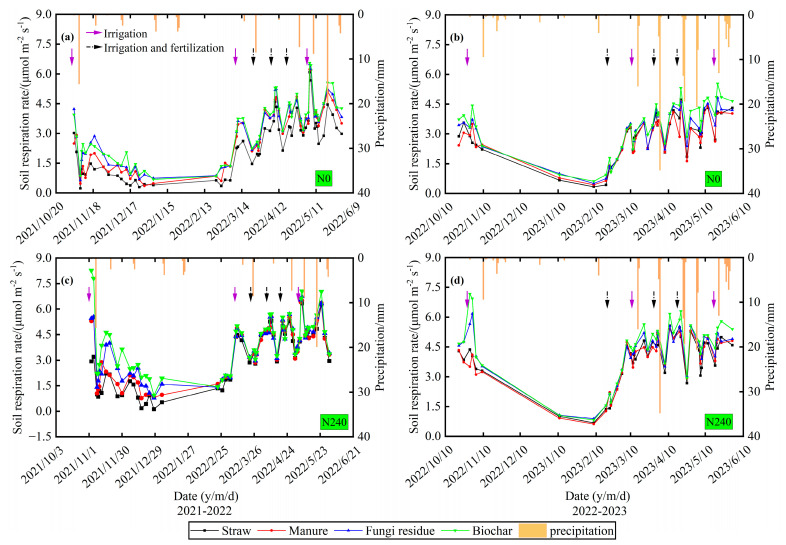
Season variation in soil respiration rates during the winter wheat growing period in the 2021–2022 season and 2022–2023 seasons. (Note: (**a**) is the soil respiration rate under N0 in 2021–2022 season; (**b**) is the soil respiration rate under N240 in the 2021–2022 season; (**c**) is the soil respiration rate under N240 in the 2022–2023 season; and (**d**) is the soil respiration rate under N240 in the 2022–2023 season).

The pattern of soil respiration rates in the winter wheat field was more consistent across all treatments during the two growing seasons, with soil respiration rates varying from 0.2 to 7.1 μmol (m^2^ s)^−1^ (Figure 3). Soil respiration rates tended to decrease from the sowing to the reviving stage and tended to increase after the reviving stage. Soil respiration rates peaked after irrigation or fertilization. In the 2021–2022 season, the peak of soil respiration rates peaked after the last irrigation event, and heavier rainfall significantly increased soil respiration rates. However, heavier rainfall did not increase the soil respiration rates but suppressed soil respiration rates in the 2022–2023 season. In addition, the application of nitrogen increased soil respiration, and soil respiration rates were highest in the biochar treatment and lowest in the straw treatment under N0 and N240 treatments.

**Figure 4:**
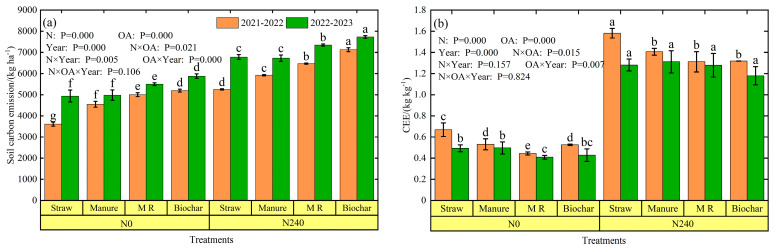
Soil carbon emission (**a**) and CEE (**b**) under different treatments during the winter wheat growing period in the 2021–2022 season and 2022–2023 season. Different lowercase letters above error bars indicate significant differences among the treatments (*p* < 0.05).

Over the two seasons, soil carbon emissions were ranked as biochar > M R > manure > straw (Figure 4a). Compared to the straw amendment, CE increased significantly by 23.49–29.6%, 14.82–22.87%, and 5.14–11.50% (*p* < 0.05) for the biochar, mushroom residue, and manure, respectively.

The M R reduced CE by 3.7–9.3% compared to the biochar amendment. Nitrogen rates had a significant effect on CEE (*p* < 0.05), but the impact of organic amendments (OAs) on CEE was inconsistent between the 2021–2022 season and 2022–2023 season (Figure 4b). The CEE of N240 was the highest in both winter wheat seasons. Compared with N0, the CEE of N240 increased by 159.3% and 173.9%, respectively, indicating that the nitrogen rate could improve CEE. In addition, the CEE of the biochar and M R treatments reduced by 7.9–21.4% and 1.6–33.5%, respectively, compared to the straw amendment.

### 3.2. SOC Stock and Ecosystem Carbon Balance under Different Treatments

**Figure 5:**
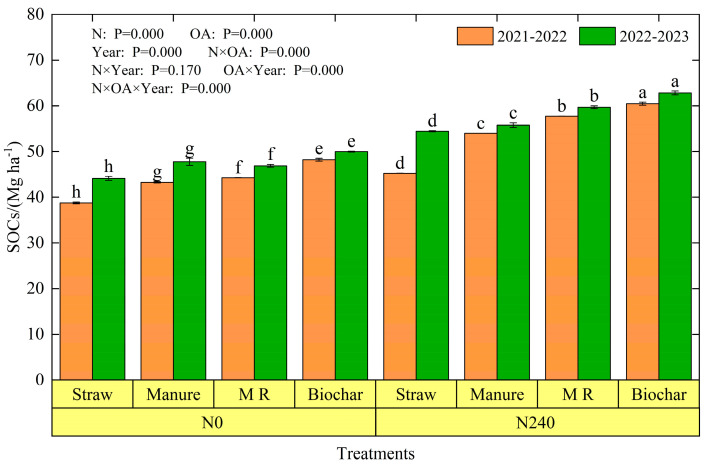
SOC stock under different treatments during the winter wheat maturity period in the 2021–2022 season and 2022–2023 season. Different lowercase letters above error bars indicate significant differences among the treatments (*p* < 0.05).

Nitrogen rates and organic amendments had a significant effect on SOC_S_ (*p* < 0.05) (Figure 5). Under the same organic amendment, the SOC_S_ of N240 was significantly higher than that of N0, with increases ranging from 16.7% to 30.5%, and the average increase was largest in the mushroom residue treatment, with a value of 28.9%, followed by the biochar treatment, with a value of 25.6%, while there was no difference between the straw and manure treatments. SOC_S_ was highest in the N240-biochar treatment, with 60.5 Mg ha^−1^ and 62.8 Mg ha^−1^ in the two seasons, and lowest in the N0-straw treatment, with 38.77 Mg ha^−1^ and 44.11 Mg ha^−1^. The SOC_S_ of the biochar, M R, and manure treatments were 13.28–33.61%, 6.27–27.61%, and 2.47–19.29% higher than those of the straw treatment, with the maximum measured in N240.

**Figure 6:**
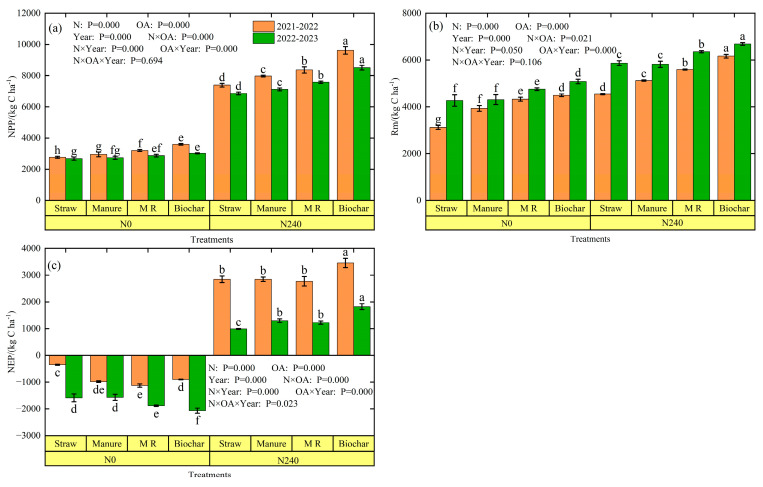
Net primary productivity (NPP) (**a**), soil microbial heterotrophic respiration (Rm) (**b**), and net ecosystem productivity (NEP) (**c**) under different treatments. Different lowercase letters above error bars indicate significant differences among the treatments (*p* < 0.05).

Net primary productivity (NPP) is an important indicator of the carbon sequestration capacity of agricultural land. Nitrogen rates, organic amendments, and the interaction between nitrogen rates and organic amendments had a significant effect on NPP (*p* < 0.05) (Figure 6a). Over the two seasons, maximum and minimum NPP were measured in the N240-biochar and N0-straw treatments, respectively. At the same nitrogen rate, the biochar, M R, and manure treatments increased NPP by 29.7–30.2%, 13.2–15.5%, and 6.5–7.9%, respectively, compared to the straw treatment. Furthermore, the NPP of N240 increased by 161.3–170.2% and 155.7–182.0% compared with N0, with the biochar treatment having the strongest carbon sequestration capacity in the 2021–2022 and 2022–2023 seasons.

NEP is the net photosynthetic productivity of ecosystems, which can be used to access the carbon balance of ecosystems. The NEP values of N0 were negative, with the N0-M R and N0-biochar treatments being the lowest for 2021–2022 and 2022–2023, respectively (Figure 6c). Under the N240 condition, organic amendments significantly affected NEP (*p* < 0.05). At the nitrogen rate of 240 kg ha^−1^, the NEP of each organic amendment was positive, indicating that nitrogen application can improve the net productivity of ecosystems. The highest NEP was observed in the N240-biochar treatment, with 3455.3 kg ha^−1^ and 1822.0 kg ha^−1^ in 2021–2022 and 2022–2023, respectively. Compared to the N240-straw treatment, the N240-biochar treatment significantly increased NEP by 21.4% and 83.9%, respectively. The N240-M R decreased NEP by 19.9% and 32.7% compared to the N240-biochar treatment. Therefore, unfertilized farmland is a source of atmospheric CO_2_, fertilized farmland is a sink of atmospheric CO_2_, and the N240-biochar treatment has the strongest carbon sink effect.

### 3.3. Yield, Biomass, PFPN, and NAE under Different Treatments

**Figure 7:**
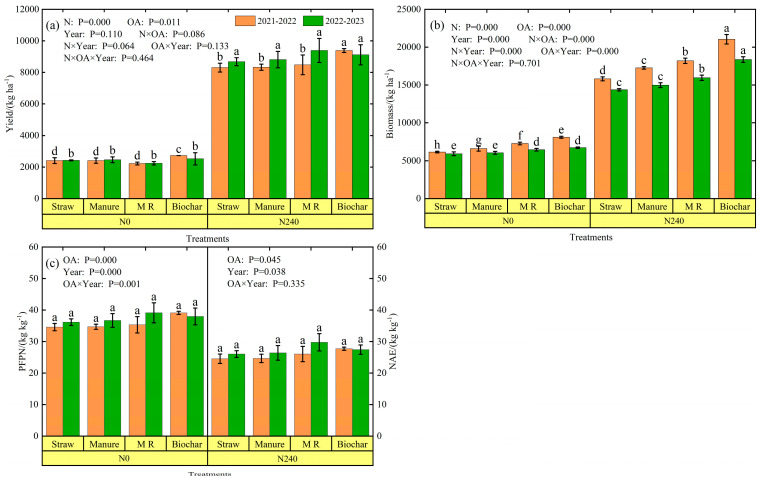
Yield (**a**), biomass (**b**), and nitrogen efficiency (PFPN and NAE) (**c**) under different treatments in the 2021–2022 season and 2022–2023 season. Different lowercase letters above error bars indicate significant differences among the treatments (*p* < 0.05).

Grain yield and aboveground biomass were higher in the N240-biochar treatment and lowest in the N0-M R treatment. Under N0 and N240 treatments, the biochar and manure treatments increased wheat grain yields by 4.0–13.3% and 0.0–1.5% compared to the straw treatment (Figure 7a). Organic amendments had a significant effect (*p* < 0.05) on wheat yields in 2021–2022, but were not significant in 2022–2023 (*p* > 0.05). Nitrogen application significantly (*p* < 0.05) increased grain yields, with the highest rate of increase in the M R treatment, at 280.83% and 318.37% in 2021–2022 and 2022–2023, respectively, under the same organic amendment; the average rate of increase was lower in the straw and manure treatments, at 250.8% and 251.3%, respectively. Over the two seasons, the interaction of organic amendments and nitrogen treatments (N0 and N240) had no significant effect on grain yield, but had a significant effect on aboveground biomass (Figure 7b).

Partial factor productivity of N (PFPN) and nitrogen agronomic efficiency (NAE) of winter wheat in the two seasons ranged from 34.59 to 39.97 kg kg^−1^ and 24.55 to 29.76 kg kg^−1^, respectively (Figure 7c). The PFPN of the biochar, M R, and manure treatments were 10.5–13.1%, 2.1–8.2%, and 0.3–1.5% higher than the straw treatment, respectively. The NAE of the biochar, M R, and manure treatments was 5.4–12.9%, 6.1–14.3%, and 0.4–1.5% higher than the straw treatment, respectively. Organic amendments significantly affected PFPN (*p* < 0.05) in 2021–2022, but were not significant in 2022–2023. The trend of NAE was different from PFPN; it was shown that biochar > M R > manure > straw in 2021–2022 and M R > biochar > manure > straw in 2022–2023, but organic amendments had no significant effect on NAE (*p* > 0.05).

### 3.4. Economic Benefits

The net profit pattern of winter wheat differed slightly between the two growing seasons (Table 4). The yield of N240 was significantly higher than that of N0 under different nitrogen rates. Under the condition of different organic amendments, the amount and price of manure and biochar are higher, and the amount and price of M R were lower, resulting in the net profit of the manure treatment being much lower than the other treatments, and the net profit of mushroom residue treatment being 1.3–16.1% higher than that of the straw treatment.

### 3.5. TOPSIS

TOPSIS was used for a comprehensive evaluation. Table 5 shows the distance between the different treatments and the positive and negative ideal scenarios and the closeness between them using the TOPSIS model. According to the final ranking, it can be seen that the N240-biochar or N240-M R ranked first or second in 2021–2022 and 2022–2023, indicating that the N240-mushroom residue treatment was the optimal treatment in this experiment.

## 4. Discussion

### 4.1. Soil Carbon Emission under Nitrogen Application and Organic Amendments

Nitrogen fertilizer is an important factor in increasing yields, but long-term overuse of nitrogen fertilizer leads to a decline in soil quality and economic efficiency and increases the soil’s contribution to atmospheric greenhouse gases, which restricts the healthy development of circular sustainable agriculture [5,6]. Organic amendments such as livestock manure, industrial wastes, and straw derivatives have a beneficial effect on soil quality and crop yields, but further research is needed due to differences in the physicochemical properties of organic amendments.

Soil CO_2_ emissions are strongly linked to temperature, moisture, and organic carbon, and these factors are influenced by the amount of nitrogen fertilizer and organic amendments [42,43,44]. In our research, the carbon emission of the N application treatment was significantly higher than that of the N0 treatment, which is consistent with the findings of Wang et al. [45]. The reason for this may be that a reduced nitrogen fertilizer application affects the crop growth, resulting in leaves not fully covering the soil surface [46], which increases soil evaporation and soil temperature but reduces soil water content, which in turn reduces carbon emissions. In addition, there are several reasons affecting CO_2_ emissions. First, N fertilization increases plant biomass, which increases soil carbon input and root respiration and stimulates soil CO_2_ emissions [47,48,49]. In our research, winter wheat biomass had a significant positive correlation (*p* < 0.05) with CO_2_ emissions in the two wheat seasons (Figure S1a), indicating that increasing biomass could stimulate CO_2_ production. Second, N fertilization further affects the rate of CO_2_ emissions by influencing soil microbial activity and function [50], which in turn affects soil carbon emissions. Nitrogen fertilization promotes microbial growth, enhances metabolic activities, and stimulates soil carbon mineralization [51], resulting in higher soil CO_2_ emissions.

Organic amendments have different effects on soil CO_2_ emissions. In this paper, the organic amendments significantly increased soil carbon emissions by 5.1–29.6% compared to straw returning, with higher emissions from mushroom residue and biochar treatments (Figure 4a). This indicates that the application of organic amendments did not suppress soil CO_2_ emissions. In previous studies, the types and amounts of organic amendments applied had different effects on soil carbon emission [45,52]. For example, both enhancement and suppression of soil CO_2_ emissions by organic amendments have been reported in different ecosystems, depending on the type and amount of organic matter [15,53]. In general, the addition of organic amendments that affect CO_2_ emissions is attributed to the input of carbon with plant and organic amendments and carbon losses due to microbial decomposition. Therefore, there are several reasons for increased CO_2_ emissions from organic amendments. Direct and indirect inputs of carbon from organic matter (by increasing crop biomass, which in turn is returned to the farmland) increase soil organic matter. Studies have shown that there is a strong relationship between soil CO_2_ emissions and organic matter levels [54]. In this experiment, soil organic carbon was significantly and positively correlated (*p* < 0.05) with total CO_2_ emissions (Figure S1b). In addition, CO_2_ emissions were closely related to microbial diversity and function [45,55]. Previous studies have shown that biochar application increases soil gram-positive and gram-negative bacteria and dehydrogenases, leading to faster soil microbial respiration, which in turn increase soil CO_2_ emissions [56,57]. Zhang et al. [58] demonstrated that straw and manure application increased soil nutrients and stimulated microbial activity, which allows the soil to maintain higher microbial biomass and enzyme activities, thus increasing soil CO_2_ emissions. This suggests that the application of organic amendments to agricultural soils affect soil microbial and enzyme activities. In this experiment, soil CO_2_ emissions from the mushroom residue treatment were significantly lower than the biochar treatment (*p* < 0.05). The reasons may be that although the C: N of the mushroom residue treatment was greater than that of the biochar treatment in this experiment, the amount of biochar applied was much greater than that of the mushroom residue, which disturbed the original carbon and nitrogen balance of the soil, and the unstable components in the biochar increased microbial abundance, leading to a higher mineralization of organic carbon than that of the mushroom residue treatment [59,60].

### 4.2. SOCs under Nitrogen Application and Organic Amendments

In this study, soils with nitrogen fertilizer application significantly increased soil organic carbon stock by 16.7–30.5% (*p* < 0.05) (Figure 5), which suggests that nitrogen inputs contribute to soil carbon stock. Previous studies have shown that the amount of nitrogen fertilizer and the way it is added can affect soil organic carbon content [61,62]. For example, N application can cause an increase, decrease, or have no effect on soil organic carbon, which is closely related to the amount and type of N applied and soil type [63,64,65]. In other words, N fertilizer affects the balance between C input and soil C loss and determines soil organic carbon stocks [66]. In general, the effect of N application on soil carbon storage can be attributed to crop biomass and microbial capacity to decompose straw and degrade existing soil carbon sources [46,67]. Therefore, N fertilization can increase carbon storage for the following reasons: N application increases the N concentration in plant tissues to increase chlorophyll content, enhances the activity of photosynthesis-related enzymes in the plant, and thus improves photosynthetic efficiency [68,69]. Furthermore, N application significantly increased aboveground biomass (*p* < 0.05) (Figure 7b), increased crop leaf area, and improved CO_2_ assimilation efficiency. On the other hand, microorganisms play an important role in the soil carbon cycle. Under the conditions of soil nutrient deficiency, microorganisms produce enzymes to promote the decomposition of organic matter and maintain their own growth, and they expand energy in the process of producing enzymes, which slows down microbial carbon fixation and leads to organic carbon loss [61,70]. Nitrogen acquisition enzyme activity decreased after N fertilizer application, and there was a negative correlation between N acquisition enzyme activity and organic carbon [61], which is a good indication of the importance of N fertilizer application on soil carbon sequestration. At present, soil organic carbon is not saturated under straw return conditions and soil carbon sequestration is not able to achieve organic carbon enhancement [7]. Application of organic amendments not only provides exogenous carbon, but also increases plant biomass, which in turn increases the input of carbon with crop biomass (Figure 7b). In this research, there was a significant difference in SOCs among different organic amendments, with biochar treatments having the highest SOCs (Figure 5), which is similar to the findings of Cheng et al. (2024) [71]. This can be explained by the fact that the application of biochar reduced carbon cycle enzyme activities [17]. The protection of organic carbon by biochar is related to the presence of its own difficult-to-break down compounds [20]. However, in this research, the enhancement of SOCs by biochar was not as high as previously reported, which may be due to the fact that the amount of biochar applied in this study (1665 kg ha^−1^ and 3286 kg ha^−1^) was much lower than that in other studies (10 t ha^−1^) [17] or that the carbon content of the biochar (260.8 g kg^−1^) was lower than that in other studies (683.1 g kg^−1^) [72]. Meanwhile, manure and mushroom residues were also able to increase SOCs compared to straw, and the SOCs of mushroom residue was higher than that of manure, which is similar to Xu et al. (2022) [7]. The difference is that the SOCs in my research is higher than the value of this research, while the percentage of carbon stock enhancement by organic amendments is lower. There may be two reasons for this: firstly, the application of the amount of manure and mushroom residues in our research were much lower than in other studies [7], resulting in a lower carbon sequestration capacity. Secondly, it may be related to soil basal nutrients; when the soil basal nutrient content is low, the soil microbial community function decreases [73] and is unable to metabolize the added carbon, so the organic amendments increase the SOCs to a greater extent.

In addition, it has been shown that SOC and SOCs are correlated with the C: N of organic amendments, i.e., organic amendments with a high C: N have a higher carbon sequestration capacity than those with a low C: N [74]. However, in our research, the SOCs of the organic amendment with high C: N (mushroom residue) was not greater than that of the organic amendment with low C: N (biochar), but it was greater than that of the organic amendment with low C: N (manure). Therefore, we believed that the C: N of organic amendments are not indicative of their carbon sequestration capacity, and that differences in the chemical structure of different organic amendments need to be considered.

### 4.3. Yield under Nitrogen Application and Organic Amendments

Leaf area index (LAI) and aboveground biomass are key factors in crop growth. Studies have shown that winter wheat yield is closely related to LAI and aboveground biomass [37]. A higher leaf area index is beneficial for generating higher aboveground biomass, thereby increasing winter wheat yield [75]. In our study, LAI and aboveground biomass were significantly greater in the N240 treatment than in the N0 treatment (Table S1, Figure 7b and Figure S2). The yield of N240 was significantly higher than N0 due to higher LAI and aboveground biomass. The yield of the N240 treatment was increased by 243.55–280.83% and 257.14–318.37% compared to N0 during the 2021–2022 and 2022–2023 growing seasons, respectively. In addition, the application of soil amendments affected crop growth and yield differently [76,77]. The addition of organic amendments can increase aboveground biomass and crop yield [78], but some researchers have also shown that the application of organic amendments reduced crop yield [79]. In this paper, biochar and mushroom residue treatments had relatively larger yield increases compared to straw treatment under different nitrogen fertilizers (Figure 7a), which may be attributed to the fact that the application of organic amendments improves the soil organic carbon content and stimulates the microbial utilization of soil nitrogen, which in turn promotes the growth of the crop and achieves the effect of a yield increase. In this paper, the increase in PFPN and NAE of both mushroom residue and biochar treatments could explain the efficient utilization of nitrogen in winter wheat.

Secondly, yield components, such as spike number, 1000-grain weight and kernels per spike, play an important role in determining grain yield, which is influenced by growing environment and management practices [80,81]. Previous studies have shown that N fertilizer application modulates yield components [80,82]. In our study, N fertilizer application significantly increased spike number, kernels per spike, and number of effective spikes per plant in winter wheat at harvest (Figure S3a–c), which is in agreement with the results of Lu et al. [80]. Yield increased with the increase of spike number, which was consistent with the results that spike number had a positive effect on yield [83]. Meanwhile, in this paper, nitrogen fertilizer application reduced 1000-grain weight (Figure S3d), but the loss from thousand-grain weight could be compensated by the increase of spike number and kernels per spike (Figure S3a,b). In addition, in this paper, under the same nitrogen fertilizer condition, compared with straw return, organic matter had no significant effect on the spike number of wheat (Figure S3a), but significantly increased the number of kernels per spike and the number of effective spikes per plant of winter wheat. This may be the limiting factor for the significant increase in yield with organic amendments.

### 4.4. The Balance of Economic and Ecological Benefits under Nitrogen Application and Organic Amendments

In this study, organic amendments increased crop yield, with higher yields under nitrogen application conditions with mushroom residue and biochar treatments (Figure 7a), which is supported by several studies [6,84]. In practice, however, the application of organic amendments on farmland is still limited by the additional economic costs involved [25].

In this study, the main factors affecting economic efficiency were yield, cost of organic amendments, labor costs, and machinery costs. The results of the 2-year experiment showed that under nitrogen fertilizer application conditions, mushroom residue provided the highest economic returns at the lowest cost compared to manure and biochar treatments (Figure 7b and Table 3). Moreover, mechanized application of organic amendments is not widely used [7]. In most cases, organic amendments are still applied manually, with labor costs accounting for 23.3–38.6% of total expenditure (Table 3). Thus, the labor cost of applying organic amendments is a major cost for small-hold farmers. To increase the widespread use of organic amendments on farms, mechanization of organic amendments could be pursued to reduce labor costs, or the use of cheaper organic amendments to reduce costs.

Net ecosystem productivity (NEP) is the difference between the amount of carbon removed from the atmosphere by photosynthesis and the amount of carbon lost through plant and soil respiration [85]. When NEP is positive, it indicates that the ecosystem is a carbon sink, while when NEP is negative, it indicates that the system is a carbon source. Our research showed that organic amendments failed to enhance soil carbon sequestration without nitrogen fertilization, whereas with nitrogen fertilization, all organic amendments were able to enhance CO_2_ sequestration, and the biochar treatment was significantly higher than the other treatments (*p* < 0.05). It has been shown that abiotic (nitrogen deposition) and biotic factors (plant height, leaf area, biomass, etc.) are closely related to net primary productivity (NPP) [86]. In this study, there were significant differences in aboveground biomass and grain yield between the organic amendment treatments, and thus in net primary productivity (NPP) between the treatments. In addition, soil heterotrophic respiration (Rm) is another factor affecting NEP, which is strongly influenced by temperature and moisture. When soil moisture is moderate, increased soil temperature increases soil CO_2_ release; when moisture is deficient or supersaturated, increased soil temperature does not increase soil CO_2_ emissions [87]. In this study, the highest soil heterotrophic respiration was observed in the biochar treatment, which may be the result of biochar increasing the soil surface temperature and microbial activity, which in turn increases CO_2_ emissions.

TOPSIS can accurately and effectively evaluate the pros and cons of planning schemes, providing reliable evaluation results [88]. Mehmood et al. [88] selected grain yield, GWPI, WPC, EWPC, and GWP as evaluation indicators, assigned weights based on their importance, and combined them with the TOPSIS method to provide the optimal strategy for increasing yield and reducing emissions in winter wheat. Zhong et al. [88] optimized using one or more objective decision-making methods to achieve the goal of reducing GWP and maintaining relatively high yields. In our research, we used the TOPSIS method and combined multiple indicators (winter wheat growth indicators, organic carbon stocks, carbon emissions, NEP, and related indicators) for effective evaluation, and the final results showed that under nitrogen application conditions, the mushroom residue or biochar amendments can balance economic benefits and soil carbon sequestration capacity.

## 5. Conclusions

This study was based on a two-year experiment conducted from October 2021 to June 2023 to measure winter wheat yield, biomass, soil carbon emissions, and soil organic carbon stocks and related metrics calculated from these metrics and analyzed using mathematical methods.

Organic amendments improved wheat yield, soil carbon stock, and soil carbon emissions. Meanwhile, net ecosystem productivity (NEP) and net profit was greater in biochar and mushroom residue with N240, respectively, compared with straw returning. The TOPSIS method showed that mushroom residue under nitrogen application conditions was suitable for application to farmland.

In the future, we will continue to analyze the applicability of different organic amendments in the North China Plain from the perspective of soil aggregate stability, soil nutrients, water and nitrogen use efficiency, soil carbon and nitrogen related genes, etc., providing basic support for the large-scale application of organic amendments.

## Figures and Tables

**Table 1:** Soil chemical properties of the 0–20 cm layer in the experimental station at the beginning of the experiment.

Treatments	Exchangeable Potassium	Available Phosphorus	Total Nitrogen	Soil Organic Carbon
	mg kg^−1^	mg kg^−1^	mg g^−1^	g kg^−1^
N0	287.07	23.61	0.79	8.89
N240	364.76	21.11	1.04	11.32

**Table 2:** The application of organic amendments (OAs) for the 2021–2022 and 2022–2023 seasons.

Nitrogen	Organic Amendments	2021–2022	2022–2023
N0	Manure/(kg ha^−1^)	2453.7	3797.5
	Mushroom residue/(kg ha^−1^)	196.3	303.8
	Biochar/(kg ha^−1^)	1665	2576.9
N240	Manure/(kg ha^−1^)	4842.4	5708.5
	Mushroom residue/(kg ha^−1^)	387.4	456.7
	Biochar/(kg ha^−1^)	3286	3873.7

**Table 3:** Detailed market price of agricultural inputs and outputs in wheat production.

Components	Unit	Price	Input Value
		CNY Unit^−1^	Unit ha^−1^
Labor	hour	20	225
Winter seed	kg	7	15
N fertilizer	kg	2.9	240
P fertilizer	kg	1.2	120
K fertilizer	kg	3.8	105
Manure	kg	0.5	9334.6
Fungi residue	kg	0.2	1680.2
Biochar	kg	0.5	5700.8
Insecticide	kg	150.0	1.1
Herbicides	kg	550.0	0.6
Fungicides	kg	820.0	1.4
Machinery	kg	2100.0	1.0
Grain	kg	2.9	225.0

Note: CNY is Chinese Yuan; the average of price and input values for the 2021–2022 season and 2022–2023 season.

**Table 4:** The economic benefits of winter wheat under different treatments during the 2021–2022 and 2022–2023 growing seasons.

Growing Season	Treatments		Input Values of Consumable Items								Output Values of Grain Yield/CNY ha^−1^	Output/Input	Net Income/CNY ha^−1^
			/CNY ha^−1^										
			Seed	Irrigation	Fertilizer	Labor	Pesticide	Machinery	Organic Amendment	Total			
					
2021–2022	N0	Straw	1575	487	1667	4200	1697	2025	0	11,651	7521	0.7	−4130
		Manure	1575	487	1667	4200	1697	2025	2454	14,105	7522	0.5	−6583
		Mushroom residue	1575	487	1667	4200	1697	2025	196	11,848	6945	0.6	−4903
		Biochar	1575	487	1667	4200	1697	2025	1665	13,316	8524	0.6	−4792
	N240	Straw	1575	487	3180	4200	1697	2025	0	13,164	25,902	2.0	12,738
		Manure	1575	487	3180	4200	1697	2025	4842	18,007	25,980	1.4	7973
		Mushroom residue	1575	487	3180	4200	1697	2025	387	13,552	26,449	2.0	12,897
		Biochar	1575	487	3180	4200	1697	2025	3286	16,450	29,285	1.8	12,834
2022–2023	N0	Straw	1575	487	1695	4800	1697	2175	0	12,429	6461	0.5	−5967
		Manure	1575	487	1695	4800	1697	2175	3798	16,226	6559	0.4	−9667
		Mushroom residue	1575	487	1695	4800	1697	2175	304	12,733	5969	0.5	−6763
		Biochar	1575	487	1695	4800	1697	2175	2577	15,006	6718	0.5	−8287
	N240	Straw	1575	487	3437	4800	1697	2175	0	14,171	23,085	1.6	8914
		Manure	1575	487	3437	4800	1697	2175	5709	19,879	23,425	1.2	3546
		Mushroom residue	1575	487	3437	4800	1697	2175	457	14,628	24,975	1.7	10,347
		Biochar	1575	487	3437	4800	1697	2175	3874	18,045	24,241	1.3	6196

**Table 5:** Distance between treatments and positive and negative ideal solutions as well as their closeness in the 2021–2022 and 2022–2023 seasons.

Nitrogen	Organic Amendment	2021–2022				2022–2023			
		D+	D−	GI	Rank	D+	D−	GI	Rank
N0	Straw	0.129	0.061	0.321	5	0.154	0.029	0.159	5
	Manure	0.147	0.037	0.199	8	0.169	0.015	0.081	8
	Fungi residue	0.143	0.040	0.221	7	0.162	0.022	0.122	6
	Biochar	0.134	0.048	0.265	6	0.166	0.019	0.104	7
N240	Straw	0.042	0.202	0.827	2	0.031	0.154	0.835	3
	Manure	0.040	0.183	0.819	3	0.050	0.138	0.733	4
	Fungi residue	0.032	0.201	0.864	1	0.019	0.167	0.896	2
	Biochar	0.050	0.222	0.815	4	0.018	0.167	0.902	1

Note: The distance between the evaluation object and the positive and negative ideal solutions is represented by D+ and D−. GI is the relative proximity: closer to 1 means that the solution is more ideal, and closer to 0 means that the solution is worse.

## Data Availability

Data will be made available on request.

## Supplementary Materials

The following supporting information can be downloaded at: https://www.mdpi.com/article/10.3390/plants13172428/s1, Figure S1: The relationship of aboveground biomass and soil carbon emission (a) and SOCs and soil carbon emission (b) in 2021–2022 and 2022–2023; Figure S2: Leaf area index (LAI) of winter wheat stages in the 2021–2022 (a) and 2022–2023 (b) growing seasons. Different lowercase letters above error bars indicate significant differences among the treatments (*p* < 0.05); Figure S3: 1000-grain weight (a), spike number (b), kernels per spike (c) and spike length (d) on yield components of winter wheat stage in the 2021–2022 and 2022–2023 growing seasons. Different lowercase letters above error bars indicate significant differences among the treatments (*p* < 0.05); Table S1: Analysis of variance showing the effects of year, nitrogen, organic amendment, and their interaction on leaf area index (LAI) of winter wheat stages in the 2021–2022 and 2022–2023 growing seasons.
