# Supplementary material for: Selection of Suitable Organic Amendments to Balance Agricultural Economic Benefits and Carbon Sequestration
Source: Plants (Basel). 2024 Aug 30;13(17):2428. doi: 10.3390/plants13172428 (PMC11397000; doi:10.3390/plants13172428)
Supplement: Supplementary file 1 [file plants-13-02428-s001.zip › plants-3132554-supplementary.pdf]

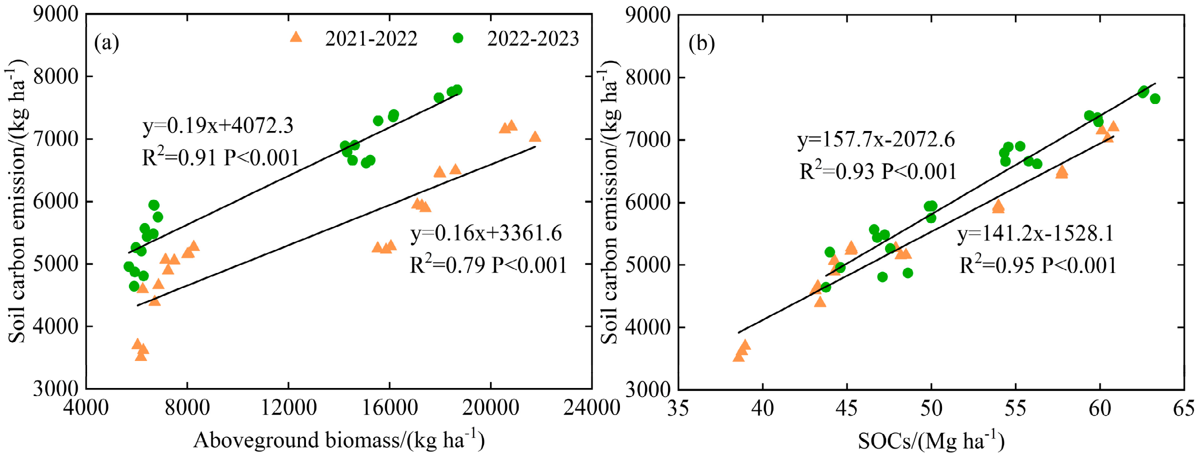

Figure S1. The relationship of aboveground biomass and soil carbon emission(a), and SOCs and soil carbon emission(b) under 2021-2022 and 2022-2023.

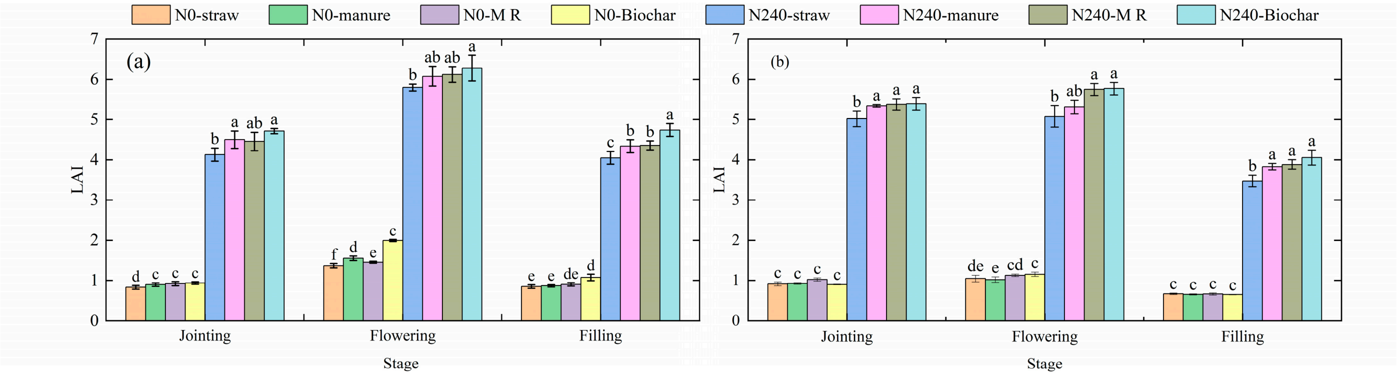

Figure S2. Leaf area index (LAI) of winter wheat stage in 2021-2022(a) and 2022-2023(b) growing seasons. Different lowercase letters above error bars indicate significant differences among the treatments ($P < 0.05$).

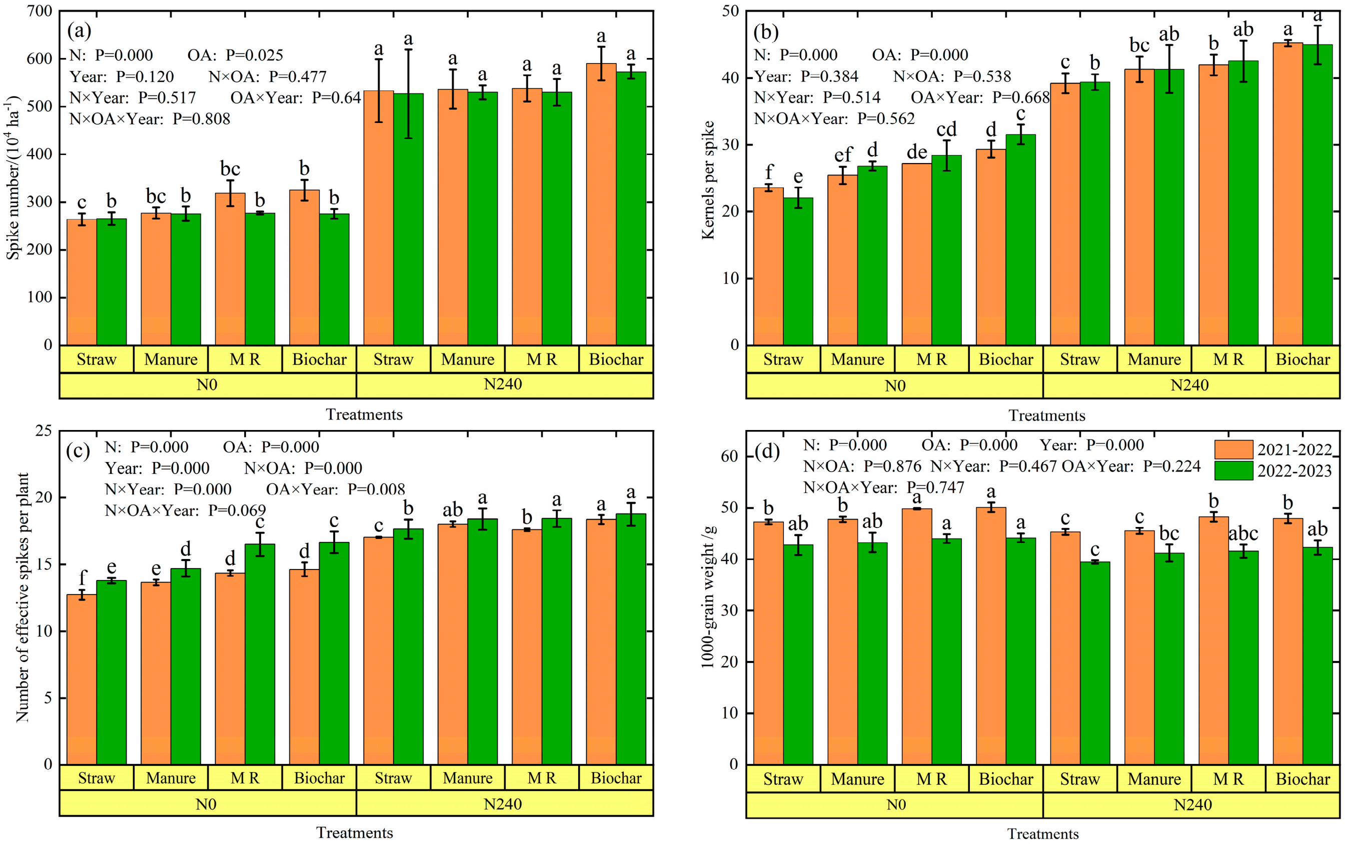

Figure S3. 1000-grain weight(a), Spike number(b), Kernels per spike(c) and Spike length(d) on yield components winter wheat stage in 2021-2022 and 2022-2023 growing seasons. Different lowercase letters above error bars indicate significant differences among the treatments (P < 0.05).

Table S1

Analysis of variance showing the effects of year, nitrogen, organic amendment and their interaction on leaf area index (LAI) of winter wheat stage in 2021-2022 and 2022-2023 growing seasons.

ANOVA	Jointing	Flowering	Filling
Year	0.000	0.000	0.000
Nitrogen	0.000	0.000	0.000
Organic amendments (OA)	0.000	0.000	0.000
Year*Nitrogen	0.000	0.348	0.000
Year*OA	0.180	0.055	0.081
OA*Nitrogen	0.000	0.023	0.000
Year*Nitrogen*OA	0.926	0.042	0.789
